# Simultaneous ipsilateral distal radius and radial head fractures: Two case reports of radius bipolar fracture

**DOI:** 10.1097/MD.0000000000024036

**Published:** 2021-01-22

**Authors:** Il-Jung Park, Yoo Joon Sur, Jongmin Kim, Jin Hwa Jeon, Ho Youn Park

**Affiliations:** a Department of Orthopaedic Surgery, Bucheon St. Mary's Hospital; b Department of Orthopaedic Surgery, Uijeongbu St. Mary's Hospital, College of Medicine, The Catholic University of Korea.

**Keywords:** distal radius fracture, proximal radius fracture, radial length, radius bipolar fracture

## Abstract

**Rationale:**

Distal radius fracture with simultaneous ipsilateral radial head fracture is a very rare pattern of injury. This type of injury is referred to as ‘radius bipolar fracture’. Treatments for this injury pattern can be challenging because both the wrist and elbow need to be considered. There are currently no guidelines for the treatment of this specific type of injury. We report two cases of this unusual pattern of injury treated in our hospital.

**Patient concerns:**

Case 1 was a 78-year-old female patient and case 2 was a 19-year-old female patient who visited our emergency department with left elbow and wrist pain after slipping and falling.

**Diagnosis:**

Plain radiography and computed tomography revealed radius bipolar fracture. Case 1 had an AO type C3 distal radius fracture, a Mason type III radial head fracture. Case 2 had an AO type B2 undisplaced distal radius fracture and a Mason type III radial head fracture.

**Interventions:**

In case 1, open reduction and internal fixation (ORIF) was performed for the distal radius fracture and radial head replacement arthroplasty for the radial head fracture. In case 2, distal radius fracture was treated conservatively and ORIF was performed for the radial head fracture.

**Outcomes:**

Bony union as achieved in both cases. At 1-year follow-up, case 1 showed slight limited range of motion of the wrist. Case 2 showed no radius shortening and full range of motion of the wrist and elbow. The Quick disabilities of the arm, shoulder and hand score was 18 and 16, respectively.

**Lessons:**

After this type of injury, the radius length can be changed, and as a result, ulnar variance can be affected. When radial head replaced is considered, it would be better to operate on the wrist first, and then perform radial head replacement. In this way, radiocapitellar overstuffing or instability can be prevented. However, if ORIF is planned for proximal radius fracture, either the proximal or distal radius can be fixed first. Surgeons should try to preserve radial length during treatment to optimize patient outcomes.

## 1 Introduction

Distal radius fracture with simultaneous ipsilateral lateral elbow injury is a very rare pattern of injury and has been defined as ‘radius bipolar fracture’.^[1–4]^ This type injury can be caused by an axial force fall on the outstretched hand with the elbow on valgus stress. Distal radius fracture is usually displaced dorsally, and a longitudinal impaction force is transmitted proximally, causing secondary fractures of the radial head/neck. Only a few cases with this pattern of injury have been reported and the incidence rate is unknown.^[5]^ Because, there are currently no guidelines for the treatment of this specific type of injury, it can be challenging because both the wrist and elbow need to be considered. This type of fracture affects the radial length and causes changes in ulnar variance. In addition, if 1 of the fracture sites is minimally displaced, it can be overlooked in the emergency room.^[2]^ We introduce this rare pattern of injury and present the clinical implications. Ethical approval for this study was waived by the Ethics Committee of our hospital considering the nature of this study (case report). Written informed consent was obtained from the patients for publication of this case report and accompanying images.

## 2 Case reports

### 2.1 Case 1

**Figure 1:**
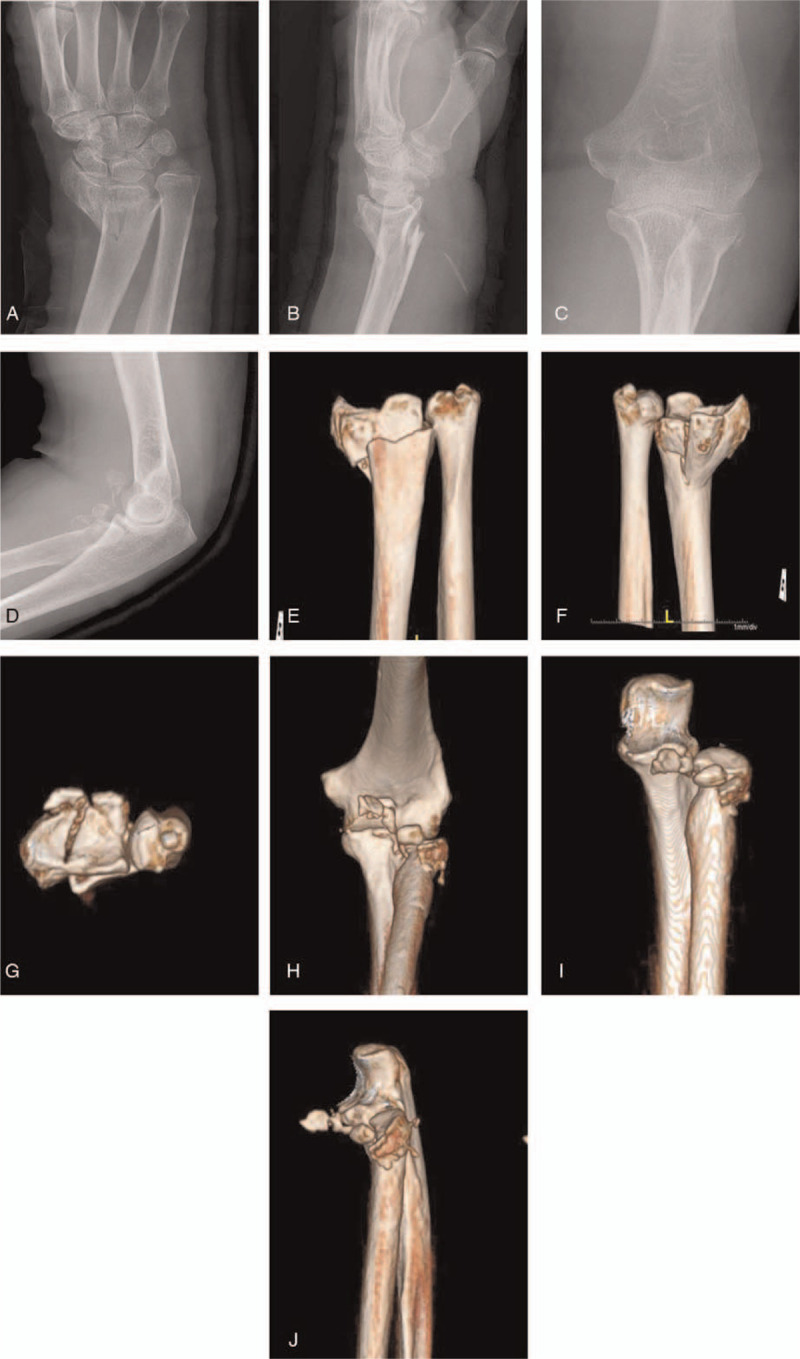
Preoperative plain radiographs of case 1. Anteroposterior and lateral X-ray images of the wrist (A, B) and the elbow (C, D) and CT images of the wrist (E-G) and the elbow (H-J) show an AO C3 distal radius fracture, a Mason type III radial head fracture, and Regan-Morrey type I coronoid process fractures.

**Figure 2:**
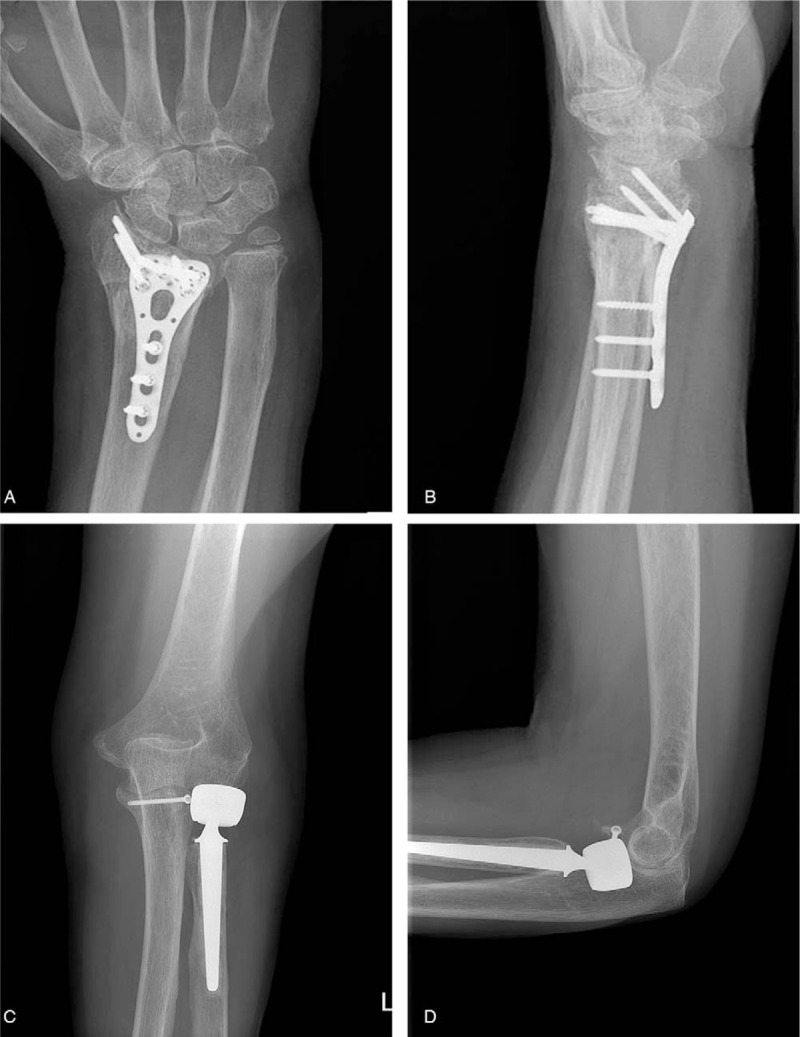
Postoperative plain radiographs of the wrist (A, B) and the elbow (C, D) of case 1 show that open reduction and internal fixation was performed for the distal radius and coronoid process fractures, and radial head replacement for the radial head fracture.

A 78-year-old female patient visited our emergency department with left elbow and wrist pain after slipping and falling. Her wrist and elbow showed swelling and painful limited ranges of motion. The neurovascular examination revealed no peripheral neurovascular injuries. The preoperative radiographs and computed tomography showed an AO type C3 distal radius fracture, a Mason type III radial head fracture, and Regan-Morrey type I coronoid process fractures. The elbow joint was not dislocated (Fig. 1). The distal radius fractures were treated with open reduction and internal fixation (ORIF) with a volar approach using a 2.4 mm Variable Angle Locking Compression Plate Distal Radius system (VA-LCP, Synthes, West Chester, PA). For the radial head fracture, radial head replacement arthroplasty (Tornier SA. Saint-Ismier, France) was performed because of the patient's age and severely comminuted fragments. The coronoid process fracture was fixed with a 3.5 mm cortical screw through the lateral approach (Fig. 2). Postoperative immobilization was applied, and an above-elbow splint was used for 2 weeks, after which a short arm splint was applied until postoperative 4 weeks. Elbow motion was allowed after postoperative 2 weeks, and wrist motion was allowed after postoperative 4 weeks. The bony union of the distal radius was visualized on x-ray at postoperative 6 weeks. The patient was followed for 13 months, at which her wrist showed 45 degrees of flexion and extension, full supination, and pronation. Her elbow showed near full range of motion. Quick Disabilities of the Arm, Shoulder and Hand score was 18 at the final follow-up visit. There were no major complications such as infection, loss of reduction, or nonunion. The x-ray showed 5.5 mm radial shortening.

### 2.2 Case 2

**Figure 3:**
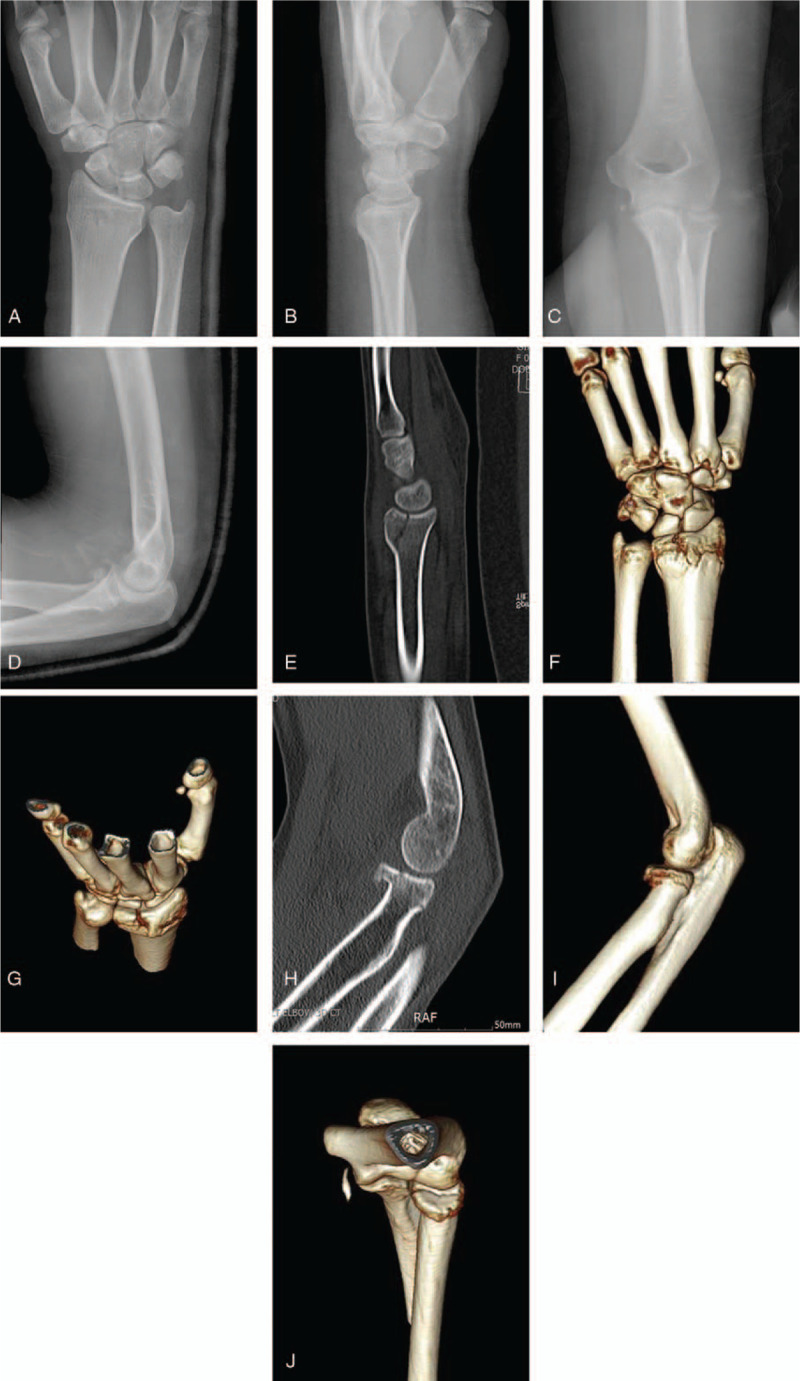
Preoperative plain radiographs of case 2. (A, B) Anteroposterior and lateral X-ray images of the wrist (A, B) and the elbow (C, D) and CT images of the wrist (E-G) and the elbow (H-J) show an AO B2 undisplaced distal radius fracture and Mason type III radial head fracture.

**Figure 4:**
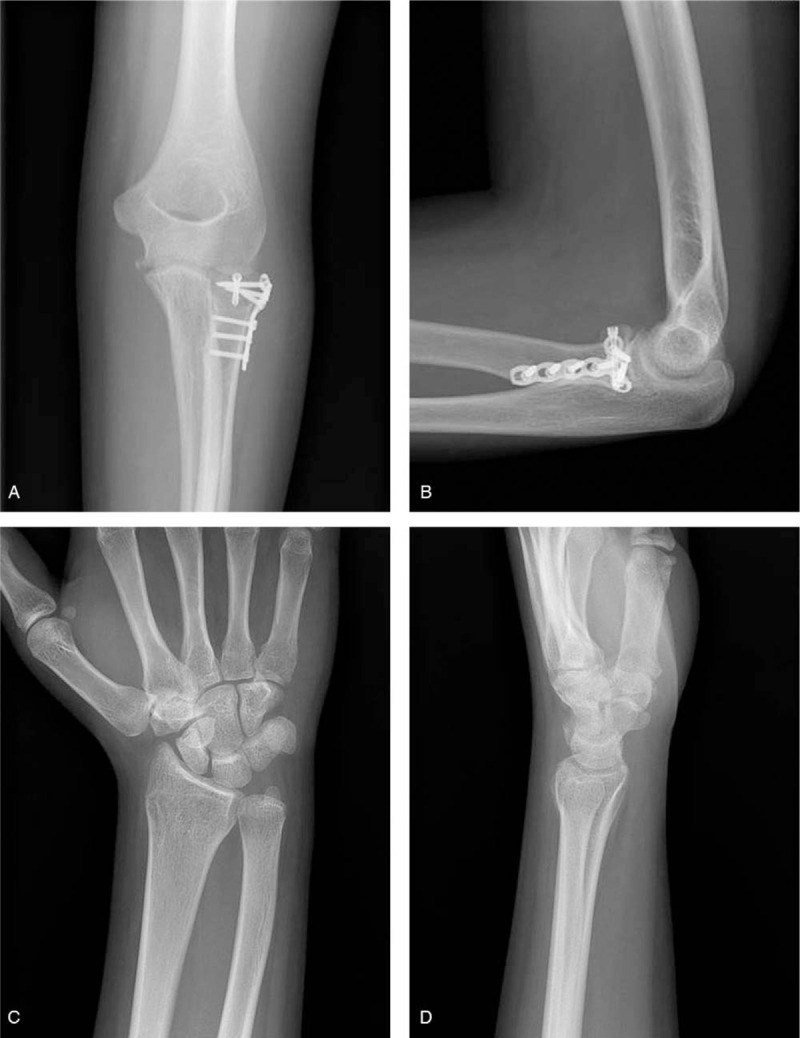
Postoperative plain radiographs of the elbow (A, B) of case 2 show that open reduction and internal fixation was performed for radial head fracture. Distal radius fracture was treated conservatively and the final plain radiographs show good bony union (C, D).

A 19-year-old female patient visited our emergency department with left elbow and wrist pain after slipping and falling. Her wrist and elbow showed swelling and painful limited range of motion, and neurovascular examination revealed no peripheral neurovascular injuries. The preoperative radiographs and computed tomography showed an AO type B2 undisplaced distal radius fracture and a Mason type III radial head fracture (Fig. 3). The distal radius fracture was undisplaced and treated conservatively. An ORIF with 2.4 mm LCP plates was performed for the radial head fracture (Fig. 4). Postoperative immobilization was performed with an above-elbow splint for 3 weeks, after which a wrist brace was applied until postoperative 4 weeks. Elbow motion was allowed after postoperative 3 weeks, and wrist motion was allowed after postoperative 4 weeks. The bony union of the distal radius was visualized on x-ray at postoperative 4 weeks. The patient was followed for 14 months, at which time her wrist and elbow showed full range of motion. Quick disabilities of the arm, shoulder and hand score was 16 at the final follow-up visit. There were no major complications such as infection, loss of reduction, or nonunion. The x-ray showed no change in ulnar variance.

## 3 Discussion

The authors introduce 2 similar cases that may have resulted from a similar injury mechanism. However, the 2 cases demonstrate that recovery can depend on patient age, bone quality, fracture pattern, and treatments type. Case 2 was a very young patient, so ORIF was performed to preserve the radial head. On the other hand, the old patient (case 1) had osteoporosis, and because both distal and proximal radius showed severe comminution, an excision of the radial head was an alternative treatment option. However, due to comminution in this case, we felt that replacement was a better option to correct and prevent future proximal migration of the radius.^[6]^ Proximal migration is associated with a positive ulnar variance and ultimately causes chronic wrist pain.

Case 2 initially reported little discomfort of the wrist because it showed minimal displacement. Distal radius fracture was found in the routine forearm and wrist radiographs performed in the emergency room during elbow fracture evaluation. Diagnostic evaluation of these injuries must be thorough due to the high incidence of missed injuries.^[2]^ The radiographs must always include the elbow and wrist joints. There should also be greater emphasis on clinical examination of the elbow in cases of wrist injuries and vice versa.

There are currently no guidelines for treatment of this specific type of injury. Management depends on fracture pattern, degree of displacement, stability of the fracture, patient's age, and physical demand of the individual patient.^[7,8]^ Based on our experiences, we would like to provide recommendations for the treatment of this specific type of injury. First, in the case of distal radius fractures associated with radial head/neck fractures, the radial length should be considered. Radial shortening can occur in proximal radius fractures, which can be more severe if accompanied by a distal radius fracture.^[9]^ Therefore, when treating a patient similar to case 1, it was recommended to operate the wrist first and then perform radial head replacement. Fixation of the distal radius allows restoration of radial length and prevents radiocapitellar overstuffing or instability. However, if ORIF is performed for proximal radius fracture, either the proximal or distal radius can be fixed first. Second, surgery and postoperative care should be performed with consideration of both the elbow and wrist. Especially in older and osteoporotic patients, early rehabilitation may lead to complications such as fracture collapse or metal failure. Many patients experience arthritic changes, and long immobilization periods can lead to joint contracture.^[10]^ Therefore, good clinical outcomes including bony union can be achieved with full explanation of prognosis and a guidance for effective rehabilitation methods.

The purpose of this report is to increase awareness of the presence of a double injury in the forearm. The present study highlights the importance of detailed clinical examination, high index of suspicion of double injury of the forearm, and radiological examination of the involved limb including both proximal and distal joints. Finally, surgeons should try to preserve radial length during treatment to optimize patient outcomes.

## Author contributions

**Conceptualization:** Il-Jung Park, Ho Youn Park.

**Data curation**: Ho Youn Park.

**Supervision:** Ho Youn Park.

**Validation:** Yoo Joon Sur.

**Visualization**: Jin Hwa Jeon.

**Writing – original draft**: Jongmin Kim.

**Writing – review & editing**: Il-Jung Park.
